# The Impact of Preoperative Nutritional Status on the Survival of Patients With Esophageal Squamous Cell Carcinoma

**DOI:** 10.3389/fsurg.2021.752792

**Published:** 2021-12-20

**Authors:** Shao-bin Chen, Di-tian Liu, Yu-ping Chen

**Affiliations:** Department of Thoracic Surgery, Cancer Hospital of Shantou University Medical College, Shantou, China

**Keywords:** esophageal neoplasm, nutrition, prognosis, squamous cell carcinoma, surgery

## Abstract

**Background:** The goal of this study was to investigate the impact of different nutritional parameters in patients with esophageal squamous cell carcinoma (ESCC) who underwent surgical resection.

**Methods:** A total of 620 patients with ESCC who underwent esophagectomy were analyzed. A receiver operating characteristic curve was constructed to set the appropriate cutoff points for five nutritional parameters: serum albumin (SA), body mass index (BMI), geriatric nutritional risk index (GNRI), prognostic nutritional index (PNI), and a new modified nutritional risk index (mNRI). Survival analyses were performed to calculate overall survival and investigate the independent prognostic factors.

**Results:** The median preoperative BMI, SA, GNRI, PNI, and mNRI values were 20.90, 42.75, 102.95, 51.90, and 63.90, respectively. The corresponding optimal cutoff points were 18.75 for BMI, 43.05 for SA, 98.5 for GNRI, 51.45 for PNI, and 61.45 for mNRI. All nutritional parameters were significantly correlated with tumor length and pT category. Decreased nutritional parameters were significantly correlated with poor survival in univariate analysis; however, only the mNRI was an independent prognostic factor in multivariate analysis (*P* = 0.041).

**Conclusions:** Nutritional parameters are convenient and valuable prognostic factors in ESCC patients who undergo surgical resection. The new mNRI parameter may be superior to the other nutritional parameters.

## Introduction

Esophageal carcinoma is a common digestive system malignancy. Esophagectomy remains the most important tool for treatment in resectable cases. Malnutrition is often observed in patients with digestive system malignancies, especially esophageal cancer. Almost 90% of the patients with esophageal cancer reported dysphagia as their major symptom at diagnosis, which could lead to reduced food intake and therefore impact the nutritional status of these patients (1). Moreover, esophageal cancer leads to increased energy consumption, which can also contribute to malnutrition (2).

Preoperative nutritional status has been found to be correlated with postoperative complications and outcomes in patients with malignancies (3, 4). Recently, various preoperative nutritional parameters have been identified as tumor biomarkers, such as body mass index (BMI), serum albumin (SA), geriatric nutritional risk index (GNRI), and prognostic nutritional index (PNI) (5–8). Previous studies found that these nutritional parameters might be correlated with survival in patients with esophageal squamous cell carcinoma (ESCC) (9–12). However, the results are still controversial (13), and few studies have evaluated the predictive accuracy among the different nutritional parameters in patients with ESCC who underwent esophagectomy.

In this study, we investigated the value of different nutritional parameters in patients with ESCC who underwent surgical resection and aimed to determine a parameter that is more convenient and valuable in clinical practice.

## Patients and Methods

### Patients

A total of 817 patients with esophageal cancer underwent esophagectomy at Shantou University Medical College Cancer Hospital between September 2014 and December 2017. Only patients with ESCC who chose surgery as their initial treatment were included in this study. This study was approved by the Ethics Committee of our hospital and conformed to the Declaration of Helsinki. Written informed consent was signed for all patients.

### Pre-operative Examinations

After the medical history taking and physical examinations, chest radiograph, barium meal, Doppler ultrasound examination of the supraclavicular lymph nodes, and contrast enhanced computed tomography scan of the chest and abdomen were routinely administrated to patients to evaluate the clinical stage of the tumor. Endoscopic ultrsonography (EUS) was also performed after the year 2010. Positron emission tomography (PET) was not routinely performed before surgery.

### Data Collection

All clinicopathological data and laboratory data were obtained from the patients' medical records. The stage of the tumor was classified based on the 8th edition American Joint Committee on Cancer TNM staging system for ESCC. Weight, height, lymphocyte counts, and SA were collected within 1 week before surgery. BMI was calculated as follows: (weight, kg)/(height^2^, m^2^). The GNRI was calculated as (1.489 × SA, g/l) + (41.7 × present/ideal body weight) (14). The PNI was calculated as 10 × SA (g/dL) + 0.005 × total lymphocyte count (per mm^3^). As the GNRI was too complicated to calculate, we tried to create a new modified nutritional risk index (mNRI) which could integrate SA and body weight in a simple pattern. As the SA and BMI have been identified as tumor biomarkers in previous studies, we set the new mNRI as SA (g/l) + BMI, and try to compared the value of this new integrated parameter to previous.

### Surgery

Most of the patients underwent esophagectomy through a right thoracotomy, while other patients underwent a left thoracotomy. For lymphadenectomy, the regional lymph nodes in the middle mediastinal, lower mediastinal, and upper abdominal regions were routinely dissected for all patients. For patients who underwent esophagectomy through a right thoracotomy, the lymph nodes around the left and right recurrent laryngeal nerves were also dissected.

### Statistical Analyses

Categorical variables were compared by the χ^2^ test or Fisher's exact test. Overall survival (OS) was calculated using the Kaplan-Meier method, and the differences between survival were compared by the log-rank test. All of the clinicopathological factors and nutritional parameters which were significant in univariate analyses were simultaneously included in multivariate Cox regression analyses to identify independent prognostic factors. The receiver operating characteristic curve (ROC) was conducted to evaluate the sensitivity and specificity for the 5-year OS, and the highest Youden‘s index was used to identify the appropriate cutoff points for BMI, SA, GNRI, PNI, and mNRI. *P* < 0.05 was set as significance. All statistical analyses were conducted in SPSS 20.0 software (IBM, Armonk, New York, USA).

## Results

### Patient Characteristics

Of the 817 patients with esophageal carcinoma who underwent esophagectomy between September 2014 and December 2017, 761 patients were diagnosed with ESCC. One hundred and sixteen patients who received neoadjuvant therapy were excluded from this study (including 94 cases of neoadjuvant chemoradiotherapy, 13 cases of neoadjuvant radiotherapy, and 9 cases of neoadjuvant chemotherapy). Twenty-five patients lacking any follow-up data were also excluded. Thus, 620 patients were enrolled for analysis in this study. There were 477 men and 143 women, and the median age was 61 years (range, 38–84 years). The mean number of lymph nodes dissected was 26.8 ± 11.0, and the median number was 26 (range, 6–74). Based on the 8th edition TNM staging system, 283 patients (45.6%) had pN0 disease, 207 patients (33.4%) had pN1 disease, 102 patients (16.5%) had pN2 disease, and 28 patients (4.5%) had pN3 disease. Radical resection was achieved in 594 patients (95.8%), while palliative resection was performed in 26 patients (4.2%). The postoperative morbidity rate was 8.3% (51/620), including 23 cases of pulmonary infection, 17 cases of anastomotic leak, and 11 cases of other complications. The hospital mortality rate was 0.5% (3/620).

There were 22 patients had multiple primary malignancies (including 5 patients with synchronous malignancy and 17 patients with metachronous malignancy). The most common sites for multiple primary malignancies were head and neck in 10 cases, the esophagogastric junction in five cases, the lung in three cases, the stomach in two cases, the breast in one case, and the colon in one case. A total of 172 patients receive adjuvant therapy after esophagectomy, including 31 cases of adjuvant chemoradiotherapy, 53 cases of adjuvant chemotherapy, and 88 cases of adjuvant radiotherapy.

### Selection of the Optimal Cutoff Point for BMI, SA, GNRI, PNI, and mNRI

The median preoperative BMI, SA, GNRI, PNI, and mNRI were 20.90 (range, 13.30–32.70), 42.75 (range, 32.30–53.20), 102.95 (range, 78.80–133.10), 51.90 (range, 35.50–75.20), and 63.90 (range, 49.10–89.40), respectively. We further used the ROC curve to determine the appropriate cutoff points for BMI, SA, GNRI, PNI, and mNRI. The areas under the curve (AUCs) for OS were 0.544, 0.562, 0.566, 0.542, and 0.567 for BMI, SA, GNRI, PNI, and mNRI, respectively. The corresponding optimal cutoff values were 18.75 for BMI, 43.05 for SA, 98.5 for GNRI, 51.45 for PNI, and 61.45 for mNRI. Furthermore, we divided the patients into two groups based on the cutoff points of each nutritional parameter as follows: low-BMI group (≤18.75) or high-BMI group (>18.75); low-SA group (≤43.05) or high-SA group (>43.05); low-GNRI group (≤98.5) or high-GNRI group (>98.5); low-PNI group (≤51.45) or high-PNI group (>51.45); and low-mNRI group (≤61.45) or high-mNRI group (>61.45).

### Correlation Between Nutritional Parameters and Clinicopathological Factors

Tables 1, 2 show patient clinicopathological factors stratified by different nutritional parameters. All nutritional parameters were significantly correlated with tumor length and pT category (*P* < 0.05). A longer tumor length and advanced pT category were more often found in the decreased nutritional parameter groups. Moreover, patients older than 60 years were more likely to have low SA, low GNRI, low PNI, or low mNRI, while male patients were more likely to have low GNRI and low mNRI. Furthermore, palliative resection was more often performed in patients with low BMI, low SA, low GNRI, or low mNRI. However, no correlations were found between the five nutritional parameters and tumor location, histologic grade, thoracotomy, or pN category.

**Table 1:** Correlation of the body mass index and serum albumin with the clinicopathological features.

	**No. patients**	**BMI**			**SA**		
		**≤18.75**	**>18.75**	***P*-value**	**≤43.05**	**>43.05**	***P*-value**
**Gender**				0.086			0.444
Male	477	113	364		265	212	
Female	143	24	119		74	69	
**Age (yr)**				0.208			<0.001
≤60	303	60	243		138	165	
>60	317	77	240		201	116	
**Tumor location**				0.622			0.206
Upper third	110	28	82		54	56	
Middle third	388	84	304		211	177	
Lower third	122	25	97		74	48	
**Tumor length**				0.044			0.022
≤5 cm	434	86	348		224	210	
>5 cm	186	51	135		115	71	
**Histologic grade**				0.164			0.287
Well	210	52	158		106	104	
Moderate	322	72	250		181	141	
Poor	88	13	75		52	36	
**Thoracotomy**				0.913			0.520
Left	161	36	125		92	69	
Right	459	101	358		247	212	
**Resection margin**				0.026			0.012
Radical	594	126	468		331	263	
Palliative	26	11	15		8	18	
**pT category**				0.039			0.026
pT1	73	12	61		33	40	
pT2	102	21	81		50	52	
pT3	373	79	294		207	166	
pT4	72	25	47		49	23	
**pN category**				0.975			0.332
pN0	283	61	222		149	134	
pN1	207	48	159		117	90	
pN2	102	22	80		61	41	
pN3	28	6	22		12	16	
**Post-operative complications**				0.336			0.017
Yes	51	14	37		36	15	
No	569	123	446		303	266	
**Adjuvant therapy**				0.664			0.712
Yes	172	36	136		92	80	
No	448	101	347		247	201	

*BMI, body mass index; SA, serum albumin*.

**Table 2:** Correlation of the GNRI, PNI, and mNRI with the clinicopathological features.

	**No. patients**	**GNRI**			**PNI**			**mNRI**		
		**≤98.5**	**>98.5**	***P*-value**	**≤51.45**	**>51.45**	***P*-value**	**≤61.45**	**>61.45**	***P*-value**
**Gender**				0.001			0.703			0.030
Male	477	154	323		223	254		156	321	
Female	143	26	117		64	79		33	110	
**Age (yr)**				0.027			<0.001			0.014
≤60	303	75	228		117	186		78	225	
>60	317	105	212		170	147		111	206	
**Tumor location**				0.478			0.650			0.423
Upper third	110	35	75		51	59		37	73	
Middle third	388	106	282		184	204		111	277	
Lower third	122	39	83		52	70		41	81	
**Tumor length**				0.003			0.001			0.004
≤5 cm	434	110	324		186	248		117	317	
>5 cm	186	70	116		101	75		72	114	
**Histologic grade**				0.753			0.648			0.796
Well	210	60	150		99	111		61	149	
Moderate	322	97	225		144	178		102	220	
Poor	88	23	65		44	44		26	62	
**Thoracotomy**				0.481			0.582			0.428
Left	161	43	118		78	83		45	116	
Right	459	137	322		209	250		144	315	
**Resection margin**				0.025			0.315			0.047
Radical	594	167	427		272	322		176	418	
Palliative	26	13	13		15	11		13	13	
**pT category**				0.009			0.006			0.007
pT1	73	17	56		24	49		17	56	
pT2	102	21	81		41	61		24	78	
pT3	373	111	262		179	194		115	258	
pT4	72	31	41		43	29		33	39	
**pN category**				0.062			0.676			0.054
pN0	283	68	215		132	151		72	211	
pN1	207	70	137		99	108		73	134	
pN2	102	35	67		46	56		37	65	
pN3	28	7	21		10	18		7	21	
**Post-operative complications**				0.094			0.030			0.083
Yes	51	20	31		31	20		21	30	
No	569	160	409		256	313		168	401	
**Adjuvant therapy**				0.833			0.771			0.388
Yes	172	51	121		78	94		48	124	
No	448	129	319		209	239		141	307	

*GNRI, geriatric nutritional risk index; PNI, prognostic nutritional Index; mNRI, modified nutritional risk index*.

For postoperative complications, only SA and PNI were found to have significant correlations. Postoperative complications were more often found in patients with low SA or Low PNI. Although postoperative complications were more often found in patients with low BMI, low GNRI, or low mNRI, the differences were not significant (*P* > 0.05).

### Survival and Prognostic Factors

The last follow-up was conducted in December 2020, with a mean follow-up time of 34.7 months (range, 1–69 months). Two hundred and fifteen patients died, and 10 patients were lost to follow-up (1.6%).

The 1-, 3-, and 5-year OS rates for all patients were 88.5, 66.0, and 61.3%, respectively. The correlations between the clinicopathological factors and survival are shown in Table 3. In univariate analysis, the variables correlated with survival were tumor length, thoracotomy, resection margin, pT category, and pN category. Patients with a tumor length ≤5 cm had a significantly improved 5-year OS than those with a tumor length >5 cm (66.2 vs. 50.0%, *P* < 0.001). Patients who underwent a left thoracotomy had a worse 5-year OS than those who underwent a right thoracotomy (53.7 vs. 64.0%, *P* = 0.005). Moreover, patients who had advanced pT category, advanced pN category, or received palliative surgery also had significantly worse survival (*P* < 0.001).

**Table 3:** Univariate analysis and multivariate analysis in regard to overall survival according to clinicopathological factors.

**Variable**	**Univariate analysis**		**Multivariate analysis**	
	**5-yr OS (%)**	***P*-value**	**HR (95% CI)**	***P*-value**
**Gender**		0.129		
Male	59.4			
Female	67.6			
**Age (yr)**		0.925		
≤60	61.1			
>60	61.6			
**Tumor location**		0.477		
Upper third	60.5			
Middle third	62.5			
Lower third	56.7			
**Tumor length**		<0.001	1.107 (0.826–1.484)	0.497
≤5 cm	66.2			
>5 cm	50.0			
**Histologic grade**		0.289		
Well	64.4			
Moderate	59.0			
Poor	63.4			
**Thoracotomy**		0.005	0.579 (0.433–0.774)	<0.001
Left thoracotomy	53.7			
Right thoracotomy	64.0			
**Resection margin**		<0.001	2.923 (1.707–5.005)	<0.001
Radical	62.9			
Palliative	21.8			
**pT category**		<0.001	1.356 (1.084–1.695)	0.008
pT1	88.2			
pT2	63.6			
pT3	61.1			
pT4	33.2			
**pN category**		<0.001	1.790 (1.548–2.070)	<0.001
pN0	75.8			
pN1	58.1			
pN2	39.4			
pN3	21.2			
**Post-operative complications**		0.378		
Yes	58.0			
No	62.9			
**Adjuvant therapy**		0.235		
Yes	63.2			
No	60.4			

*CI, confidence interval; HR, hazard ratio; MST, median survival time; OS, overall survival*.

**Figure 1:**
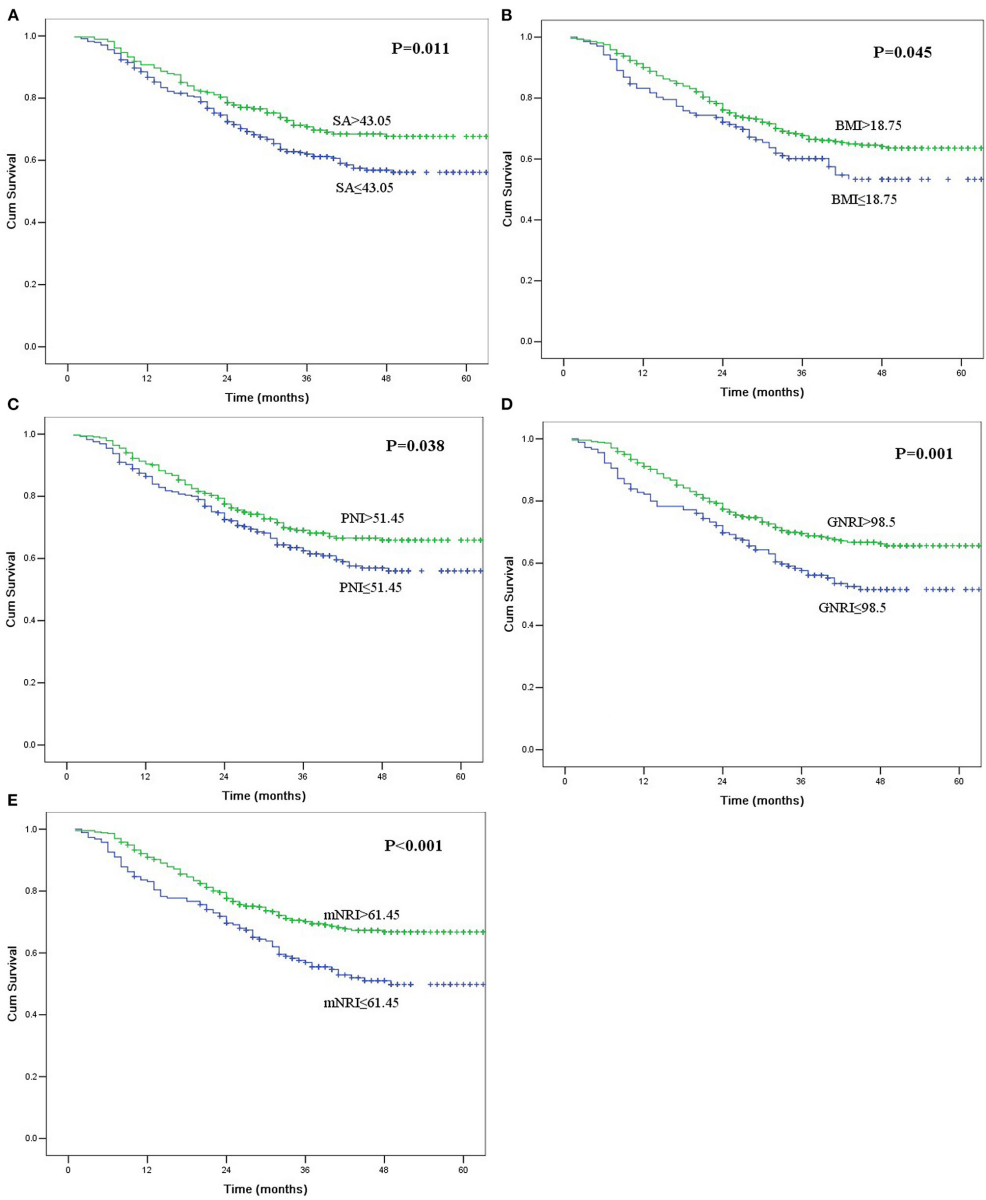
Kaplan-Meier curves for overall survival according to serum albumin (SA, **A**), body mass index (BMI, **B**), prognostic nutritional index (PNI, **C**), geriatric nutritional risk index (GNRI, **D**), and modified nutritional risk index (mNRI, **E**). All of the survival differences were significant (*P* < 0.05).

Table 4 shows the impact of the nutritional parameters on survival in univariate analysis. All five nutritional parameters were significantly correlated with survival (Figure 1). Patients in the decreased nutritional parameter groups had significantly worse survival than those in the higher groups.

**Table 4:** Univariate analysis and multivariate analysis in regard to overall survival according to nutritional parameters.

**Variable**	**Univariate analysis**		**Multivariate analysis**	
	**5-yr OS (%)**	***P*-value**	**HR (95% CI)**	***P*-value**
**SA**		0.011	0.894 (0.618–1.294)	0.554
≤43.05	56.8			
>43.05	67.6			
**BMI**		0.045	0.854 (0.590–1.235)	0.401
≤18.75	53.3			
>18.75	63.5			
**mNRI**		<0.001	0.478 (0.216–0.986)	0.041
≤61.45	49.8			
>61.45	66.7			
**PNI**		0.038	0.973 (0.696–1.359)	0.872
≤51.45	56.0			
>51.45	65.9			
**GNRI**		0.001	1.594 (0.708–3.591)	0.261
≤98.5	51.5			
>98.5	65.6			

*BMI, body mass index; CI, confidence interval; GNRI, geriatric nutritional risk index; HR, hazard ratio; mNRI, modified nutritional risk index; MST, median survival time; OS, overall survival; PNI, prognostic nutritional Index; SA, serum albumin*.

The multivariate analysis incorporated clinicopathological factors and nutritional parameters that were significant in the univariate analyses. Thoracotomy, resection margin, pT category, and pN category were independent prognostic factors in this study; however, tumor length was not an independent risk factor (*P* = 0.497). Although all five nutritional parameters were significantly correlated with survival in univariate analyses, only the mNRI value was an independent risk factor in multivariate analysis (*P* = 0.041).

## Discussion

The identification of factors associated with high-risk, prior to treatment, is important for determining an individual therapeutic strategy for patients with malignancies. Currently, the TNM staging system is widely used for predicting the outcomes of esophageal cancer and other malignancies. Although a separate clinical stage (cTNM) was provided in the 8th edition for ESCC to be used as a prognostic indicator before treatment, its predictive value is still limited. The clinical stage is mainly determined by imaging, so it may be limited by individual techniques (15). The Worldwide Esophageal Cancer Collaboration (WECC) recommended that a series of examinations be conducted to obtain a reliable cTNM stage, but not all of these modalities were available in every institution (15). Therefore, we think that it is necessary to develop other easily accessible and effective indicators to predict the outcome of esophageal cancer patients before treatment, which may help to improve individualized treatment.

Preoperative malnutrition has been reported to be a predictor of postoperative complications and outcomes in cancer patients and can be used as a prognostic indicator (16–18). Most nutritional parameters, such as ALB and BMI, can be easily obtained in clinical examinations and routine laboratory examinations. However, these parameters may be easily affected by many factors, such as inflammation or hydration status (19–21). Recently, other nutritional parameters, such as the GNRI and PNI, have been proposed to evaluate nutritional-related risk. Previous studies also found that these nutritional parameters were independent prognostic factors in different kinds of cancers (3, 8, 22, 23). However, to date, few studies have compared the prognostic accuracy of different nutritional parameters in ESCC patients.

In this study, we investigated the value of different nutritional parameters, including BMI, SA, GNRI, PNI, and a new mNRI, in ESCC patients who underwent surgical resection. We found that all nutritional parameters were significantly correlated with tumor length and pT category. A longer tumor length and advanced pT category were more often found in the decreased nutritional parameter groups. It is easy to understand that the nutritional condition for patients with esophageal cancer is correlated with tumor length and invasion depth, as patients with larger primary tumor sizes may suffer a longer period of dysphagia and more serious symptoms, which may lead to insufficient oral intake and malnutrition.

Although all five nutritional parameters were significantly correlated with survival in univariate analyses, only the mNRI was an independent risk factor in multivariate analyses in our study. Moreover, the AUC for OS of the mNRI was higher than that of the other nutritional parameters. These results indicate that the new nutritional parameter (mNRI) could be used as an indicator to evaluate the prognosis of ESCC patients who underwent surgery and might be more effective than the other nutritional parameters. Further studies are needed to elucidate the correlation between our new nutritional parameter (mNRI), other nutritional parameters and the prognosis of patients with ESCC.

The mechanism by which malnutrition correlates with the poor prognosis of cancer patients is still not clear. There are several possible explanations. First, malnutrition may be associated with immune suppression in patients, which may provide a favorable microenvironment for tumor recurrence and lead to cancer recurrence after surgery (24, 25). Second, the presence of malnutrition might decrease the tolerance and response to treatment, which might also lead to the poor prognosis of cancer patients. Andreyev et al. (26) found that the poor survival of gastrointestinal cancer patients with malnutrition undergoing chemotherapy might be a result of treatment with a lower dose. Di Fiore et al. (27) found that for patients with esophageal cancer who underwent chemoradiotherapy, a higher SA level was a predictor of a complete response. Third, preoperative malnutrition may increase postoperative complications and the mortality rate. The esophagectomy procedure is very invasive with a higher mortality rate than other gastrointestinal cancers, while pulmonary complications are one of the most important factors for perioperative mortality (28). Previous studies have found that malnutrition patients who undergo esophagectomy may develop more pulmonary complications. Kamachi et al. (29) analyzed 340 esophageal cancer patients who underwent esophagectomies and found that malnutrition increased postoperative pulmonary complications. Masoomi et al. (17) analyzed 6,352 esophageal cancer patients who underwent esophagectomies and found that weight loss was the most important factor for acute respiratory failure. Finally, malnutrition patients may have a higher risk of non-cancer death. Dignam et al. (30) found that in breast cancer, underweight patients suffered a higher rate of non-cancer death than normal-weight patients. Migita et al. (31) found that more underweight patients with gastric cancer died of non-cancer causes, especially infection, than normal-weight patients with gastric cancer. According to these theories, we think that a perioperative nutritional intervention, such as administration of immunonutrition and dietary counseling, may improved the nutritional status and tolerance to treatment in malnutrition patients with malignancies. However, more studies are needed to further elucidate the mechanism and evaluate our hypothesis.

The major limitation of this study was that it was retrospective and single-center in nature. Moreover, the AUC for OS for all five nutritional parameters was small, indicating that the diagnostic accuracy of these parameters was low. We think that multicenter studies with larger cohorts are needed to evaluate our findings in this study, especially the value of our new mNRI for patients with ESCC.

In conclusion, our study demonstrated that nutritional parameters were convenient and valuable prognostic predictors in patients with ESCC who underwent surgical resection. A new mNRI parameter might be superior to the other nutritional parameters in prognosis evaluation.

## Data Availability Statement

The raw data supporting the conclusions of this article will be made available by the authors, without undue reservation.

## Ethics Statement

The studies involving human participants were reviewed and approved by Ethics Committee of Shantou University Medical College Cancer Hospital. The patients/participants provided their written informed consent to participate in this study.

## Author Contributions

S-bC designed the research, analyzed the data, and wrote part of the paper. D-tL analyzed the data and wrote part of the paper. Y-pC designed the research and analyzed the data. All authors contributed to the article and approved the submitted version.

## Funding

This work was supported by the Medical Scientific Research Foundation of Guangdong Province of China (B2019070).

## Conflict of Interest

The authors declare that the research was conducted in the absence of any commercial or financial relationships that could be construed as a potential conflict of interest.

## Publisher's Note

All claims expressed in this article are solely those of the authors and do not necessarily represent those of their affiliated organizations, or those of the publisher, the editors and the reviewers. Any product that may be evaluated in this article, or claim that may be made by its manufacturer, is not guaranteed or endorsed by the publisher.
